# Tigecycline as a dual inhibitor of retinoblastoma and angiogenesis via inducing mitochondrial dysfunctions and oxidative damage

**DOI:** 10.1038/s41598-018-29938-x

**Published:** 2018-08-06

**Authors:** Ying Xiong, Wei Liu, Qian Huang, Jierong Wang, Yanjun Wang, Huijuan Li, Xuedong Fu

**Affiliations:** 1 Wuhan University, Zhongnan Hospital of Wuhan University, Department of Pediatrics, 430071 Wuhan, China; 2 Wuhan University, Zhongnan Hospital of Wuhan University, Department of Pharmacy, 430071 Wuhan, China

## Abstract

Retinoblastoma is the most common intraocular malignancy in children with poor prognosis. The progression of retinoblastoma is dependent on a robust angiogenic response. Targeting both retinoblastoma cells and angiogenesis may therefore provide an alternative therapeutic strategy in retinoblastoma. Here, we demonstrated the inhibitory effects of tigecycline, a FDA-approved antibiotic, in retinoblastoma and angiogenesis *in vitro* and *in vivo*. We showed that tigecycline significantly inhibited growth and induced caspase-dependent apoptosis of multiple retinoblastoma cell lines. Tigecycline also effectively inhibited angiogenesis through suppressing capillary network formation, migration, proliferation and survival of human retinal microvascular endothelial cell (HREC). Mechanistically, tigecycline acts on both retinoblastoma cells and HREC via inhibiting mitochondrial protein translation, resulting in mitochondrial dysfunction, energy crisis, and oxidative damage. Importantly, we demonstrated the *in vivo* efficacy of tigecycline in inhibiting retinoblastoma and angiogenesis, and inducing oxidative stress on xenograft mouse model. In addition, ATP levels and growth rates were largely affected in retinoblastoma ρ0 cells that lacked mitochondrial respiration. Our work provides systematic pre-clinical evidence for repurposing tigecycline from its traditional use for retinoblastoma treatment. Our work demonstrates the essential roles of mitochondrial metabolism in both retinoblastoma and its angiogenesis.

## Introduction

Retinoblastoma is the most common childhood primary intraocular cancer, which occurs in 1/15, 000 live births worldwide^1^. The clinical management of retinoblastoma is challenging and its prognosis is poor, particularly in developing countries^2,3^. Retinoblastoma is a heterogeneous disease with aberrant activation of oncogenes and suppression of the tumour suppressor genes, such as retinoblastoma 1^4^. Retinoblastoma is initiated by the mutation of the tumor suppressor gene retinoblastoma 1 (*RB1*) and characterized by molecular heterogeneity and extensive vascularization^5,6^. Angiogenesis, the growth of new blood vessels from pre-existing ones, is tremendously important to retinoblastoma growth, survival and metastasis^7^. Therefore, the ideal therapeutic strategies for retinoblastoma are to target both tumor cells and angiogenesis.

Tigecycline is a broad spectrum antibiotic that acts on bacteria through binding to ribosome and suppressing protein synthesis^8,9^. It is clinically used to treat complicated skin and intra-abdominal infections, and community-acquired bacterial pneumonia^10^. Interestingly, substantial evidence on pre-clinical models demonstrate that tigecycline is a novel type of anti-cancer drug. At clinically achievable doses, tigecycline effectively kills leukaemias, renal and liver cancer cells, and significantly augments chemotherapy agents *in vitro* and *in vivo*^11–13^. Importantly, tigecycline exhibits preferentially anti-cancer activities with minimal toxicity in normal counterparts^11,13^. The mechanisms of tigecycline in cancer cells include inhibition of mitochondrial translation and Wnt/β-catenin, and autophagy activation^11,14,15^.

In this work, we are the first to investigate the effects of tigecycline on retinoblastoma and angiogenesis. We further analysed the underlying mechanism of tigecycline and validated the results obtained from *in vitro* cell culture system on retinoblastoma xenograft mouse model. Our work show that tigecycline is effective in inhibiting retinoblastoma growth, survival and angiogenesis via inhibiting mitochondrial translation, and inducing mitochondrial dysfunctions and oxidative damage.

## Results

### Tigecycline effectively targets both retinoblastoma cells and angiogenesis

It is well known that angiogenesis plays essential roles in retinoblastoma progression and metastasis^16^. To determine the possible effects of tigecycline in retinoblastoma, we investigated not only the growth and survival of retinoblastoma cells but also angiogenesis after tigecycline treatment. Three cell lines Y79, WERI-Rb-1, and RB116 that represent *in vitro* human retinoblastoma models are used in our study. Of note, these cells express stem cell markers (eg, Nanog and Oct3/4) and retinal progenitor markers (eg, PAX6 and CHX10). In addition, Y79 and WER-Rb-1 contain RB1 mutations^17–19^.

**Figure 1:**
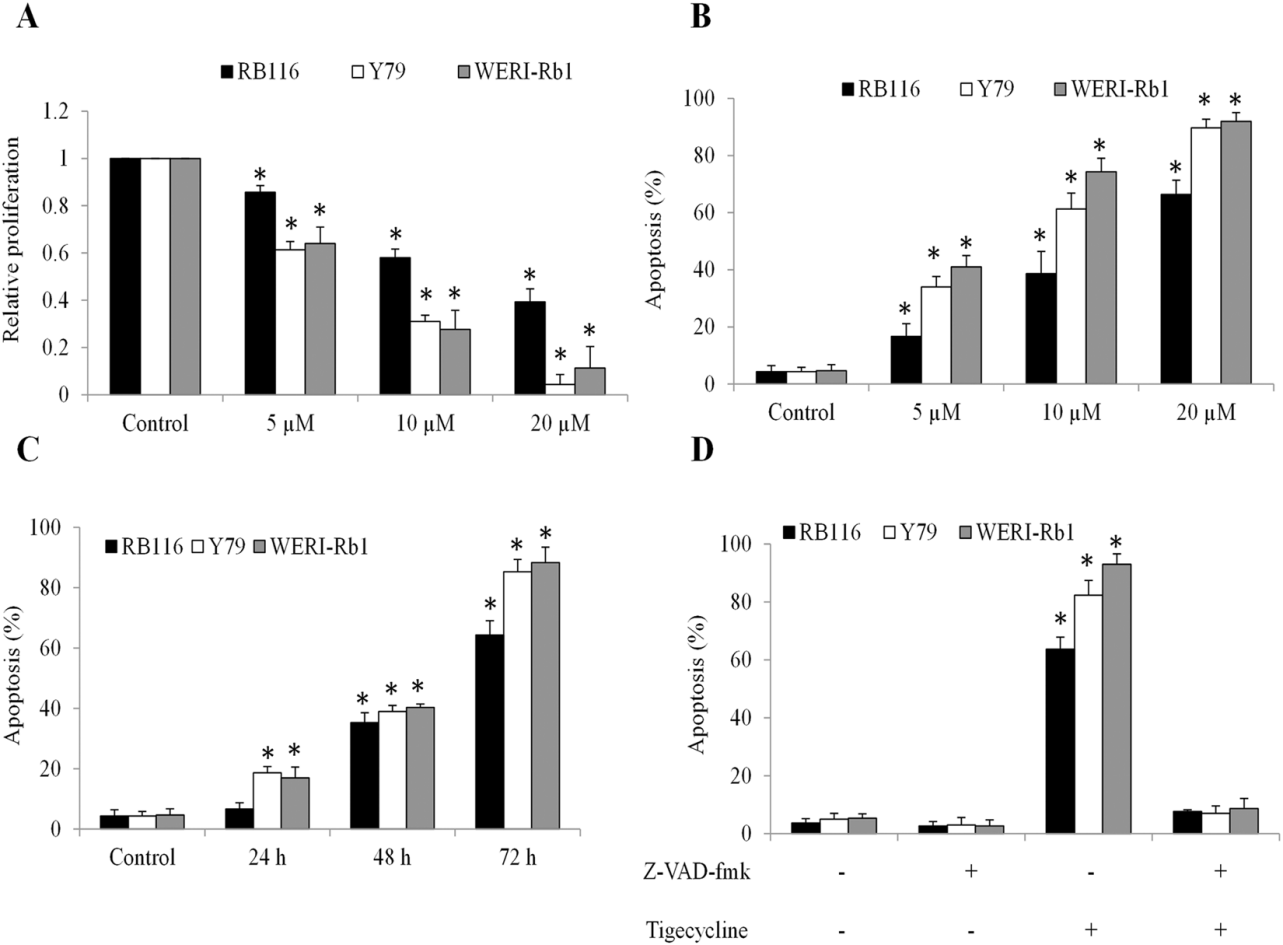
Tigecycline significantly inhibits proliferation and induces caspase-dependent apoptosis in retinoblastoma cells. Tigecycline at 5, 10 and 20 µM inhibits proliferation (**A**) and induces apoptosis (**B**) of retinoblastoma cell lines: Y79, RB116 and WERI-Rb1. (**C**) Tigecycline at 20 µM significantly induces apoptosis of retinoblastoma cells in a time-dependent manner. (**D**) Tigecycline (20 µM) is ineffective in inducing apoptosis in the presence of a pan-caspase inhibitor Z-VAD-fmk (50 µM). *p < 0.05, compared to control.

We found that tigecycline at 5 to 20 µM significantly inhibited proliferation in all tested retinoblastoma cell lines (Fig. 1A). Tigecycline also significantly increased Annexin V percentage in retinoblastoma cells in a dose- and time-dependent manner (Fig. 1B,C), suggesting that tigecycline induced apoptosis. In addition, tigecycline induced apoptosis starting from 24 hour (Fig. 1C). A pan-caspase inhibitor Z-VAD-fmk completely abolished the effects of tigecycline in inducing apoptosis (Fig. 1D), demonstrating that tigecycline induced apoptosis through activating caspase pathways.

We further demonstrated that tigecycline at 10 and 20 µM significantly inhibited proliferation and induced apoptosis in normal retinal epithelial cells, but to a lesser extent than in retinoblastoma cells (Supplementary Fig. S1). In addition, tigecycline at 5 µM significantly targeted retinoblastoma cells but did not affect normal retinal epithelial cells (Supplementary Fig. S1). Although differences in tigecycline’s efficacy on normal and tumor cells was moderate, these results demonstrated that tigecycline had preferential activity on tumor cells. This is also consistent with other work and our previous findings on the selectivity of tigecycline on tumor than normal counterparts^11,20^.

**Figure 2:**
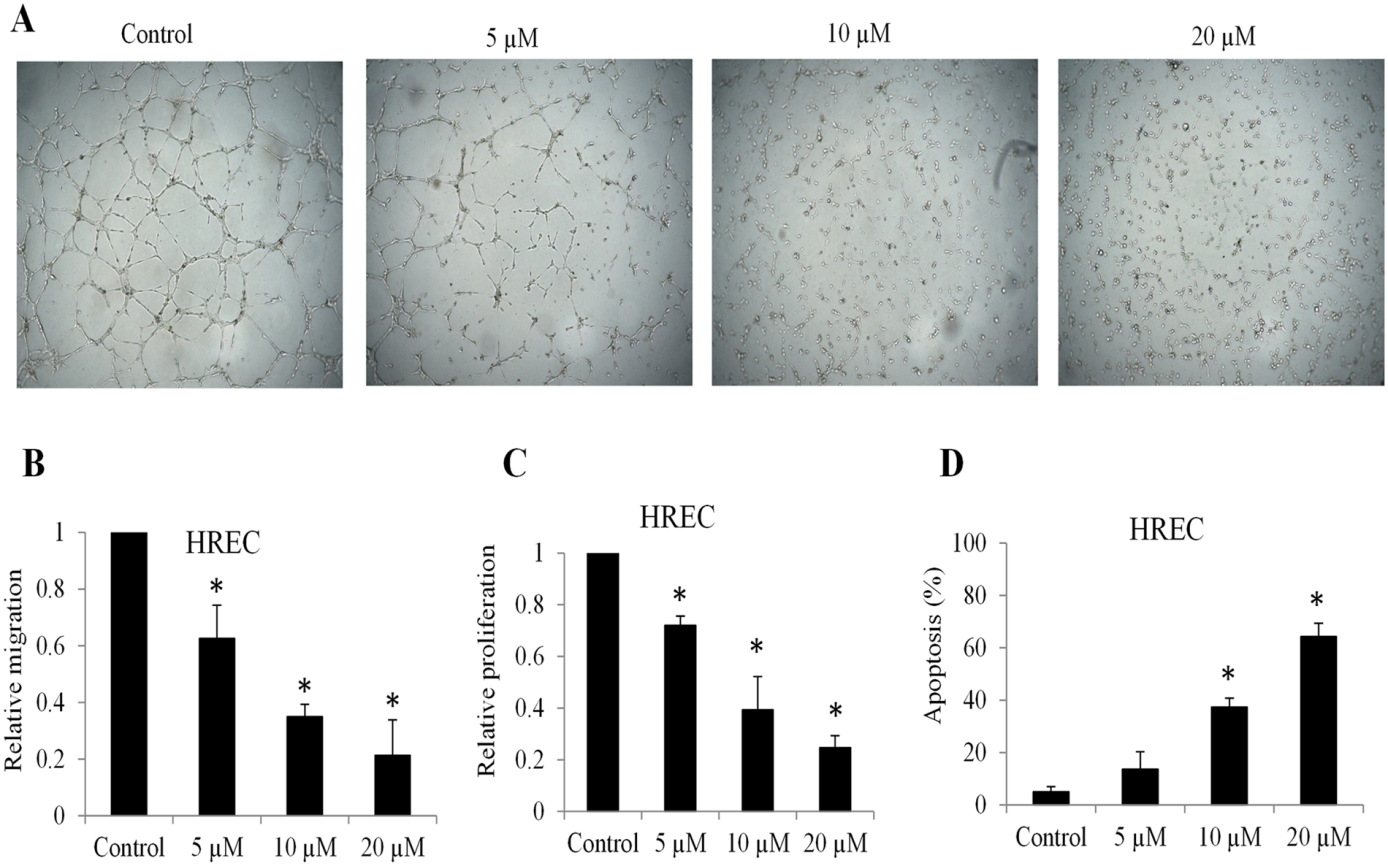
Tigecycline inhibits retinal angiogenesis. (**A**) Representative images of *in vitro* capillary network formation showing dose-dependent inhibitory effect of tigecycline on retinal angiogenesis. *In vitro* capillary tube formation was fully formed at 6 hours after plating primary human retinal microvascular endothelial cells (HREC) onto complete Matrigel. Tigecycline significantly decreases migration (**B**), proliferation (**C**) and increases apoptosis (**D**) of HRECs. *p < 0.05, compared to control.

We performed *in vitro* angiogenesis assay by plating human retinal microvascular endothelial cells (HREC) onto Matrigel gel. We observed a rapid alignment and tubular structures formation within 6 hours in control. In contrast, HREC failed to form proper capillary structures in the presence of 5 and 10 µM tigecycline. Notably, hardly any tubular network formed in the presence of 20 µM tigecycline (Fig. 2A). We further found that tigecycline significantly inhibited HREC migration and proliferation, and induced apoptosis in a dose-dependent manner (Fig. 2B–D). Time course analysis of apoptosis indicated that tigecycline at 20 µM significantly induced apoptosis of endothelial cells starting from 36 hours drug treatment (Supplementary Fig. S2). Taken together, our results demonstrate that tigecycline targets multiple aspects of biological functions of both retinoblastoma cells and HREC.

### Tigecycline inhibits mitochondrial protein translation, and induces mitochondrial dysfunctions and oxidative stress in retinoblastoma cell and HREC

**Figure 3:**
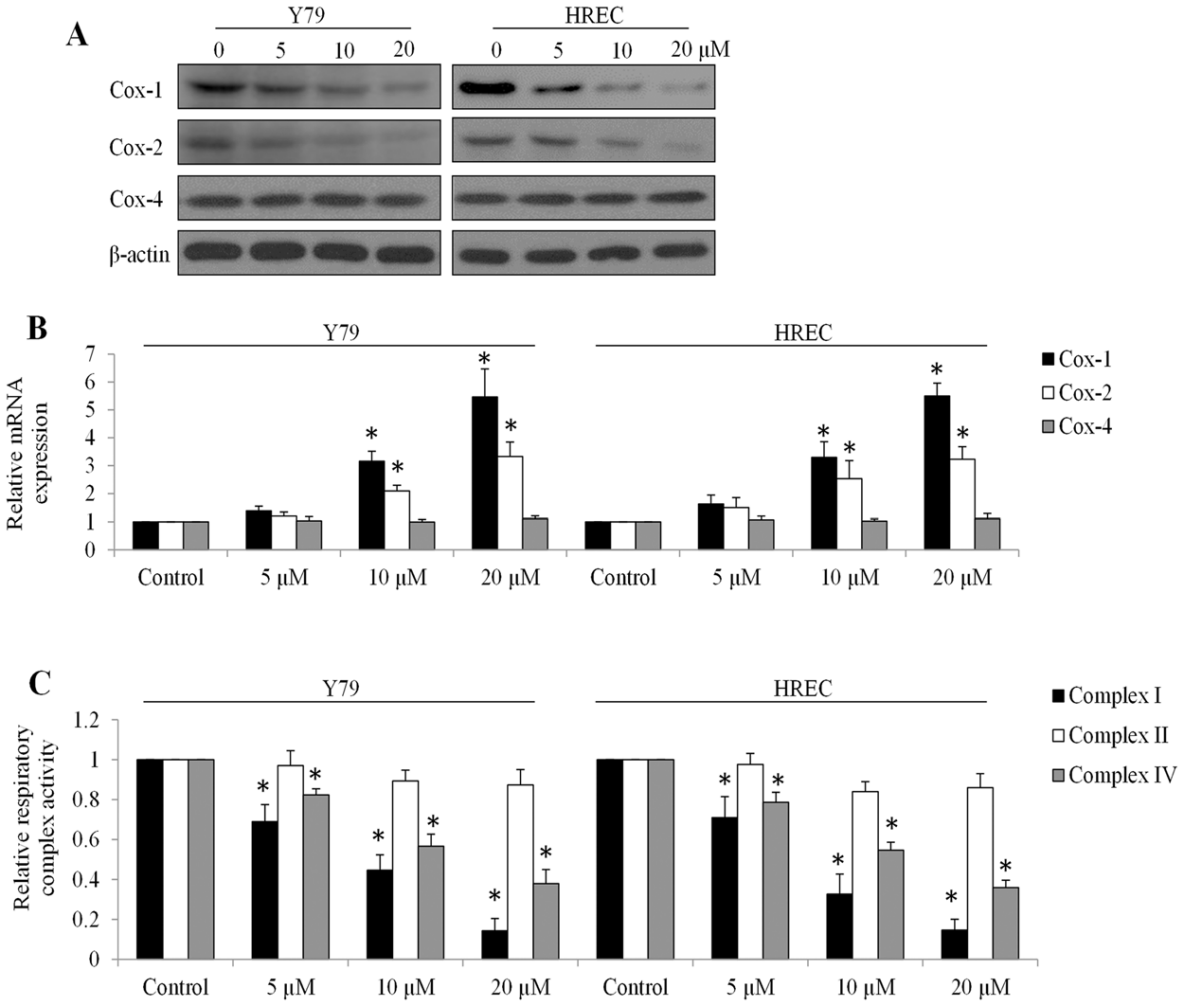
Tigecycline inhibits mitochondrial translation in retinoblastoma and HREC. Tigecycline decreases protein level (**A**) and increases mRNA level (**B**) of Cox-1 and -2 without affecting protein and mRNA levels of Cox-4 in retinoblastoma cells and HREC. Transcript levels of each gene was normalized with β-actin. (**C**) Tigecycline suppresses mitochondrial respiratory complex I and IV but not II activity in Y79 and HREC cells. *p < 0.05, compared to control.

Tigecycline acts as a protein synthesis inhibitor in bacteria^8,9^. In addition, tigecycline has been demonstrated to have potent inhibitory activity on mitochondrial protein translation in human leukaemia and renal carcinoma cells^11,13^. To determine whether the inhibitory effects of tigecycline on retinoblastoma cell and HREC is mediated by a similar mechanism, we investigated the mitochondrial translation via examining protein and mRNA levels of cytochrome c oxidase-1, 2 and 4 (Cox-1, Cox-2 and Cox-4, respectively). Although all three proteins are subunits of mitochondrial respiratory complex IV, Cox-1 and Cox-2 are mitochondrial DNA and only translated by mitochondrial ribosomes whereas Cox-4 is translated by nuclear ribosomes^21^. We observed significantly decreased protein levels and increased mRNA levels of Cox-1 and 2 in Y79 cells and HREC exposed to tigecycline (Fig. 3A,B, and Supplementary Fig. S3). In contrast, tigecycline did not affect the mRNA or protein levels of Cox-4 (Fig. 3A,B). These results clearly show that tigecycline specifically inhibits mitochondrial protein translation in retinoblastoma cells and HREC.

**Figure 4:**
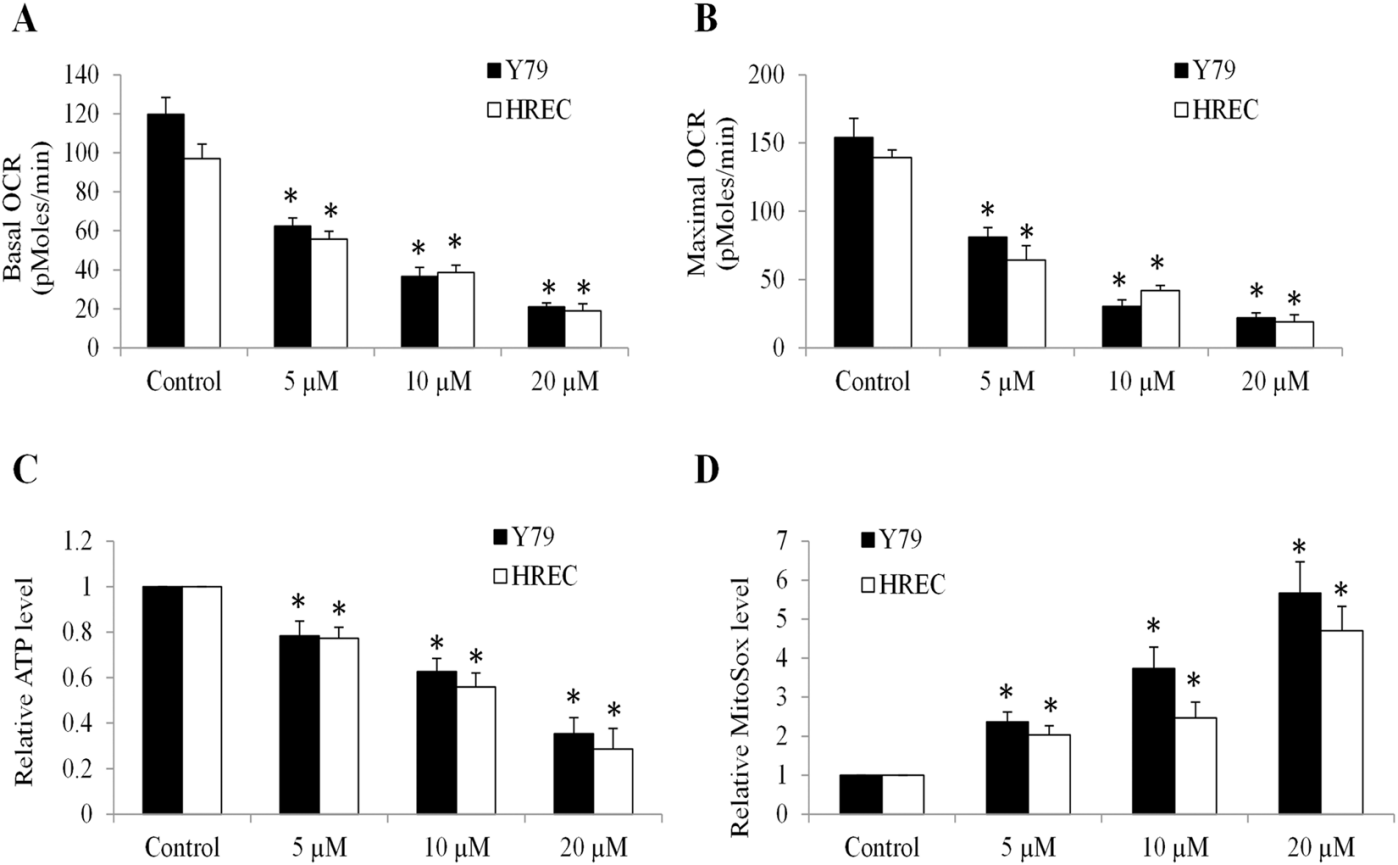
Tigecycline inhibits mitochondrial respiration and ATP production, and induces oxidative stress in retinoblastoma and HREC. Tigecycline significantly decreases basal (**A**) and maximal (**B**) OCR in a dose-dependent manner in Y79 and HREC. Tigecycline significantly decreases ATP (**C**) and increases mitochondrial superoxidase (**D**) level in Y79 and HREC. *p < 0.05, compared to control.

**Figure 5:**
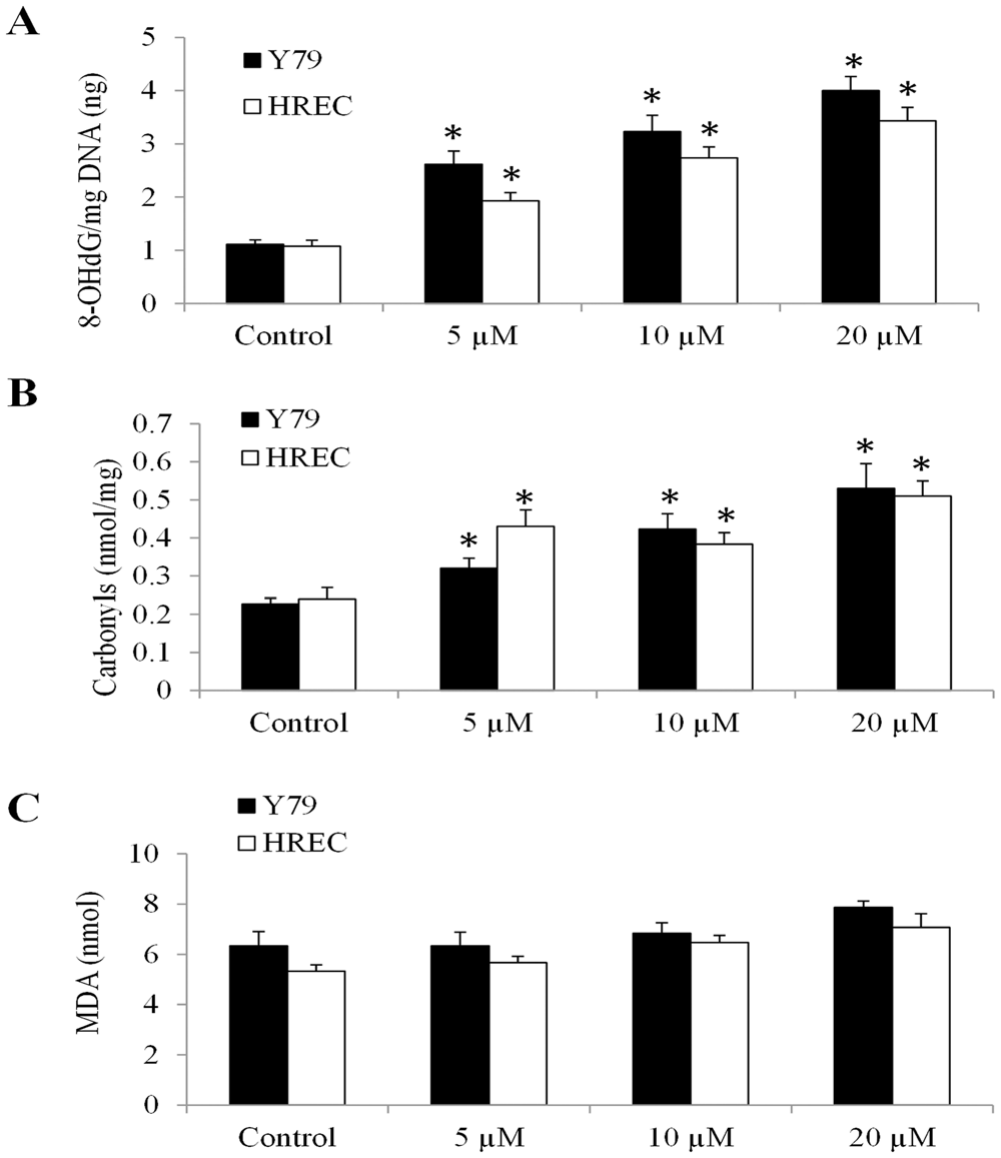
Tigecycline causes oxidative damage in retinoblastoma and HREC. Tigecycline significantly increases 8-OHdG (**A**) and carbonylation (**B**) but not MDA (**C**) level in Y79 and HREC. *p < 0.05, compared to control.

Mitochondrial respiratory complexes I and IV but not II contain mitochondrially encoded protein subunits^22^. Consistent with mitochondrial translation inhibition, tigecycline significantly inhibited activities of respiratory complexes I and IV but not II (Fig. 3C). Since mitochondrial respiration rely on the proper function of mitochondrial respiratory chains, we next analyzed the oxygen consumption rate (OCR, indicative of mitochondrial respiration). We observed the dose-dependent reduction on basal and maximal OCR levels in Y79 and HREC exposed to tigecycline (Fig. 4A,B), demonstrating that tigecycline inhibits basal mitochondrial respiration and reserved respiratory capacity. In addition, tigecycline significantly decreased ATP production and increased mitochondrial superoxidase in Y79 and HREC (Fig. 4C,D). An oxidized DNA byproduct 8-OHdG and protein carbonyls were also increased by tigecycline in Y79 and HREC (Fig. 5A,B). We did not observe significant changes on malondialdehyde (MDA, an end product of lipid peroxidation) levels in cells exposed to tigecycline (Fig. 5C). These results demonstrate that tigecycline induces mitochondrial functions and oxidative damage in retinoblastoma and HREC.

### Tigecycline is ineffective in targeting mitochondrial respiration-deficient retinoblastoma ρ0 cells

**Figure 6:**
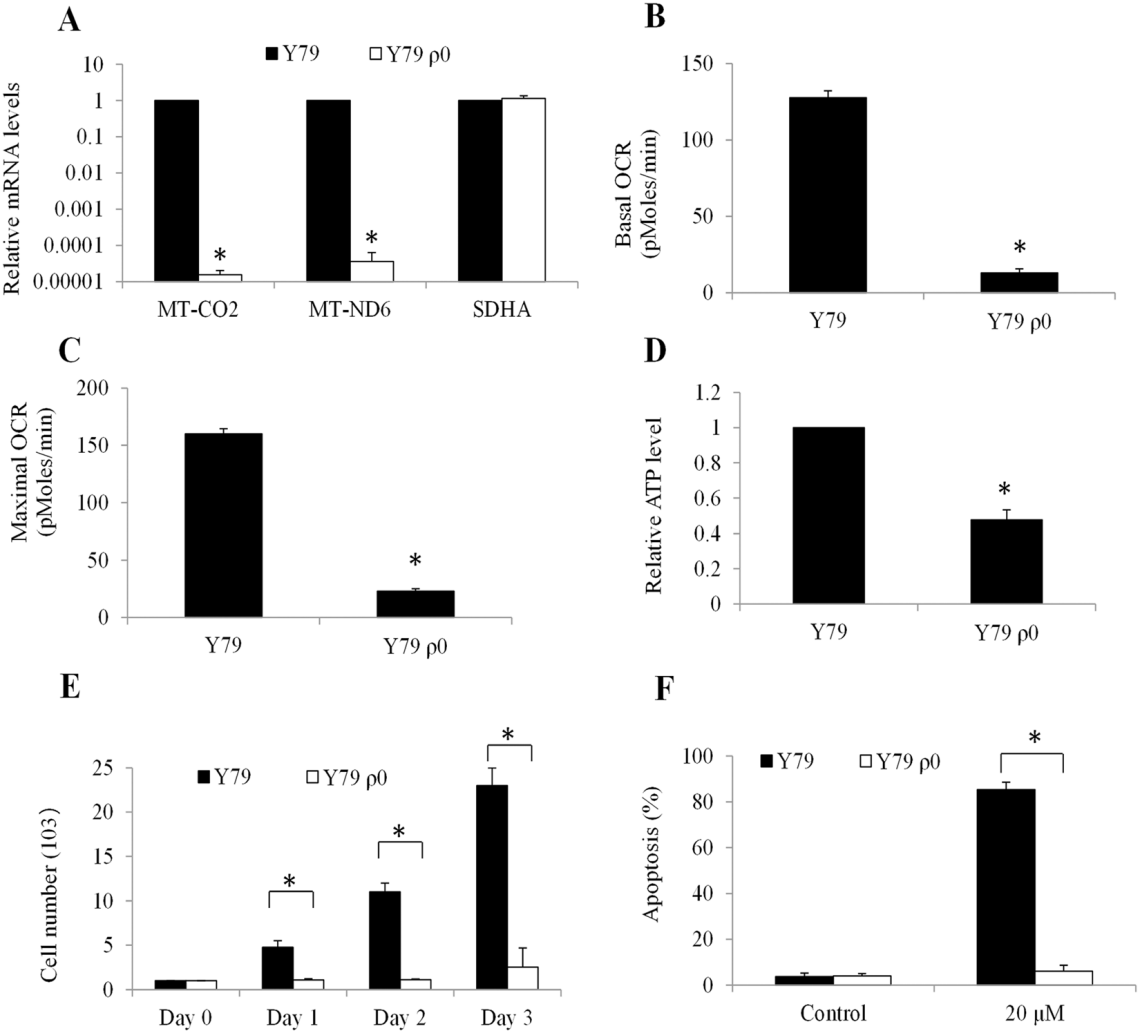
Tigecycline induces retinoblastoma cell apoptosis through its suppression of mitochondrial respiration. (**A**) Remarkable reduction on the mRNA expression of two mitochondrial genome-encoded (MT-ND6 and MT-CO2) but not nuclear genome-encoded (SDHA) respiratory chain enzyme subunits in Y79 ρ0. Remarkable reduction on the basal (**B**) and maximal (**C**) OCR level in Y79 ρ0 cells. Significant reduction in ATP levels (**D**) and minimal growth rate (**E**) in Y79 ρ0 cells. (**E**) Tigecycline (20 µM) is ineffective in inducing apoptosis in Y79 ρ0 cells. *p < 0.05, compared to Y79 wildtype.

To further confirm that tigecycline targets a mitochondrial genome-encoded component of oxidative phosphorylation in retinoblastoma, we generated Y79 ρ0 cells that lack mitochondrial DNA and are incapable of performing mitochondrial respiration^23^. We successfully established Y79 ρ0 cells from Y79 cells with the very minimal levels of mitochondrial DNA (eg, MT-CO2 and MT-ND6) without affecting nuclear DNA (eg, SDHA) (Fig. 6A). These ρ0 cells also exhibit a remarkably reduced baseline OCR and reserved respiratory capacity compared to parental cells (Fig. 6B,C). In addition, these ρ0 cells show significantly decreased ATP level and inadequate growth (Fig. 6D,E). Notably, Y79 ρ0 cells are resistant to tigecycline in inducing apoptosis (Fig. 6F). Thus, our data indicate that tigecycline specifically targets a mitochondrial genome-encoded component of mitochondrial respiration in retinoblastoma.

### Tigecycline inhibits retinoblastoma growth and angiogenesis, and induces oxidative stress *in vivo*

**Figure 7:**
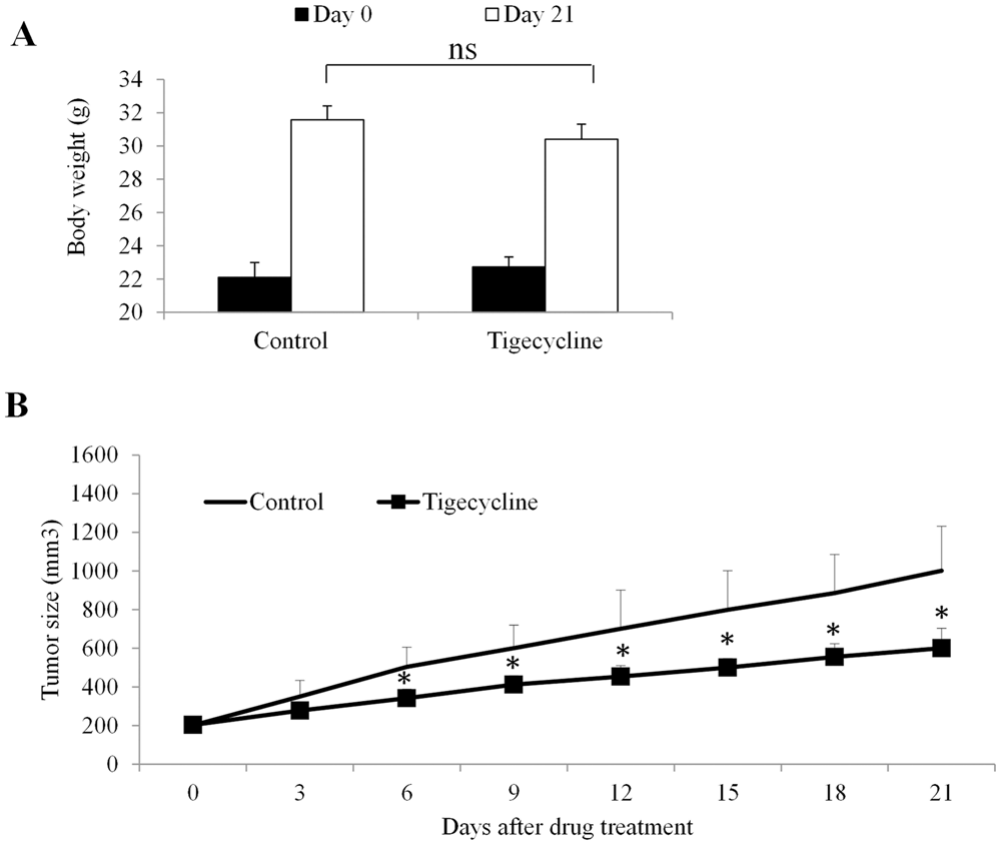
Tigecycline significantly inhibits retinoblastoma growth in mice without toxicity. (**A**) No significant changes on mouse body weight in control and tigecycline-treated groups. (**B**) Tigecycline significantly inhibits retinoblastoma growth *in vivo*. *p < 0.05, compared to control. ns, not significant.

**Figure 8:**
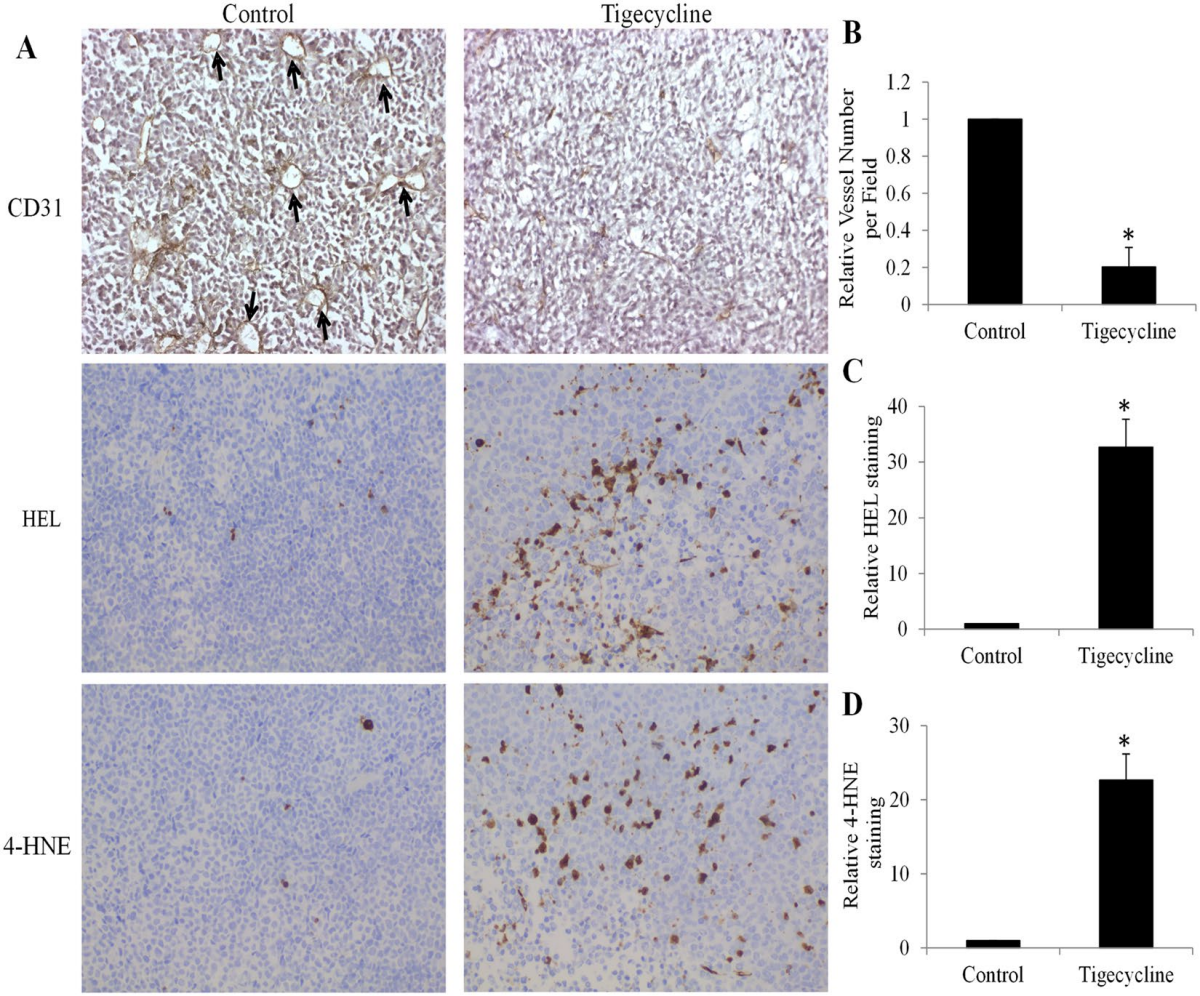
Tigecycline significantly inhibits retinoblastoma angiogenesis and induces oxidative stress *in vivo*. (**A**) Representative photos of immunohistochemistry of microvascular marker CD31, oxidative stress markers HEL and 4-HNE staining. Tigecycline significantly inhibits retinoblastoma angiogenesis (**B**) and increases oxidative stress (**C**,**D**) *in vivo*. Intraperitoneal tigecycline at 60 mg/kg once per day were given to the mice. *p < 0.05, compared to control.

To investigate whether the data obtained from *in vitro* cell culture system are reproducible in *in vivo*, we investigated the effects of tigecycline on retinoblastoma growth and retinoblastoma angiogenesis in xenograft mouse model. We also analysed the oxidative stress level in retinoblastoma tumours. We found that tigecycline at 60 mg/kg significantly inhibited retinoblastoma growth in mice throughout the whole duration without affecting the mice body weight (Fig. 7 and Supplementary Table S1), indicating that tigecycline at tolerable dose has anti-retinoblastoma activity in mice. Immunohistochemistry staining of tigecycline-treated tumour tissue sections with anti-CD31 antibody (which stains tumour ECs) revealed a decreased tumour vascularization compared with control (Fig. 8A, indicated by white arrows). The average tumour vascular density of each tumour group, which included all lumen and non-lumen CD31 structures or cells, was significantly decreased tigecycline treatment (Fig. 8B). Of note, a significantly increased staining of oxidative stress markers HEL, and 4-HNE tigecycline-treated tumour tissue sections were observed (Fig. 8A,C,D). These results were in concordance with our *in vitro* data, and confirmed the inhibitory effects of tigecycline in retinoblastoma and angiogenesis and its ability in inducing oxidative stress.

## Discussion

The majority of solid tumors, such as retinoblastoma, are largely dependent on angiogenesis to grow and spread. Histopathologically, retinoblastoma tumor shows perivascular sleeves consisting of neoplastic cells, demonstrating a close association of tumor cells with blood vessels^24^. High vascularization in retinoblastoma has been shown to be linked with chemoresistance, dissemination and poor prognosis^16,25^. In contrast, angiogenesis inhibition alone by bevacizumab or pigment epithelium-derived factor suppresses retinoblastoma growth without producing significant systemic toxicity^26,27^. Therefore, targeting angiogenesis as well as tumour cells as an alternative therapeutic strategy has been hotly investigated for the treatment of various cancers^28,29^. In lines with these efforts, we identified an antibiotic drug tigecycline as a dual inhibitor targeting both retinoblastoma and angiogenesis.

The anti-cancer activities of tigecycline has been recently revealed in various cancers, such as leukemia, cervical and breast cancer^11,14,15^. Of note, tigecycline has been moving forward in clinical trials for the treatment of acute myeloid leukaemia (ClinicalTrial No. NCT01332786) and chronic myeloid leukaemia (ClinicalTrial No. NCT02883036). Our present work adds retinoblastoma to the list of tigecycline-targeted cancers by showing the pro-apoptotic and anti-proliferative activities of tigecycline in multiple retinoblastoma cell lines as well as retinoblastoma xenograft mouse model (Figs 1 and 7). In addition, the preferential selectivity of tigecycline on cancer cells over normal counterparts demonstrates tigecycline as a promising candidate for targeted cancer therapy^11,13^. We also demonstrated the preferential activity of tigecycline on retinoblastoma cells compared to normal retinal pigment epithelial cells (Supplementary Fig. S1). Tigecycline at doses that effectively inhibit retinoblastoma tumour growth do not cause significant toxicity to mice (Fig. 7). These data agree with the previous work and suggest the therapeutic window of tigecycline on cancer treatment.

We further demonstrated that tigecycline also inhibits angiogenesis through affecting various biological functions of HREC (Fig. 2). We confirmed the inhibitory effects of tigecycline on retinoblastoma angiogenesis in xenograft mouse model (Fig. 8A,B). The inhibition of corneal neovascularization by tigecycline has been shown in a rat model^30^. Our findings further extend the previous work that tigecycline inhibits angiogenesis induced not only by chemical cauterization but also by tumor progression. Doxycycline, another tetracycline-type antibiotic has also been shown to inhibit choroidal neovascularization^31^. Our work together with the previous findings suggest the anti-angiogenic activity of tetracycline-type antibiotic. The current clinically used angiogenesis inhibitors, such as sorafenib and bevacizumab, have been shown to only have marginal benefits to cancer patients^32^. It is possibly because angiogenesis inhibitors target the blood vessels without affecting tumor cells. The ability of tigecycline or tetracycline-type antibiotic in targeting tumor and tumor angiogenesis distinguishes it from anti-cancer compounds that only target cancer cells or angiogenesis inhibitors that only target vascular endothelial cells.

Mechanistically, we show that tigecycline acts on retinoblastoma and HERC cells via inhibiting mitochondrial protein translation (Fig. 3A,B). As a consequence of mitochondrial translation inhibition, tigecycline induces mitochondrial dysfunction, energy crisis and oxidative damage (Figs 3–6). Consistent with the results obtained from *in vitro* cell culture system, we also observed the increased oxidative stress in retinoblastoma tumor exposed to tigecycline (Fig. 8), further confirming mitochondria as the target of tigecycline in retinoblastoma. Apart from mitochondrial translation inhibition, tigecycline has been reported to inhibit Wnt/β-catenin and autophagy in cervical and breast cancer, respectively^14,33^. It seems that tigecycline acts on cancer in a cancer type-specific manner, we and others show mitochondrial inhibition as the mechanism of tigecycline’s action on most cancers^11,13^. Substantial recent evidences indicate that many tumours are highly dependent on mitochondrial respiration for growth and survival^34–36^. In line with these findings, we demonstrate that mitochondrial respiration-deficient retinoblastoma cells have remarkably reduced ATP level and growth (Fig. 6D,E). Our work clearly demonstrate that mitochondrial metabolism plays an important role in retinoblastoma.

In conclusion, we provide evidence that tigecycline is a useful addition to the treatment armamentarium for retinoblastoma by demonstrating tigecycline as a dual inhibitor of retinoblastoma and angiogenesis. The toxicity and efficacy of tigecycline in clinical settings are important and warrant further investigation. Our work also highlights the therapeutic value of targeting mitochondrial metabolism in retinoblastoma.

## Materials and Methods

### Cell lines, primary cells and mitochondrial respiration-deficient ρ^0^ cell line generation

Human normal retinal pigment epithelial cell line hTERT RPE-1 and human retinoblastoma cell lines Y79, WERI-Rb-1, and RB116 (kind gifts from Dr. Zhi Li)^29^ were cultured using RPMI 1640 medium supplemented with 10% FBS, 1% HEPES (Invitrogen, US) and 100 U/mL penicillin-streptomycin (Thermo Fisher Scientific, US). Mitochondrial respiration-deficient ρ^0^ cells were generated by culturing Y79 cells in the above medium and selecting using 1 µg/ml ethidium bromide (EtBr), supplemented with 50 µg/ml uridine and 1 mM sodium pyruvate (Sigma, US) for 8 weeks^37^. Human primary retinal microvascular endothelial cells (HREC) (Cell Systems, US) were originated using Serum-Free Medium (SF-4Z0-500, Cell Systems, US), and subsequently grown and passaged in Cell Systems Complete Medium (4Z0-500, Cell Systems, US).

### MTS proliferation

Cells (5000/well in 96-well plate) were incubated with DMSO or different concentrations of tigecycline (Sigma, US) for 3 days. Proliferation was then determined by using MTS Cell Proliferation Colorimetri Assay Kit (BioVision, US).

### Annexin V labelling and flow cytometry

Cells (100,000/well in 12-well plate) were treated with DMSO or tigecycline, or Z-VAD-fmk (carbobenzoxy-valyl-alanyl-aspartyl-[O-methyl]- fluoromethylketone, Selleckchem, US). After 3 days treatment, apoptotic cells were labelled by using Annexin V-FITC and 7-AAD (BD Pharmingen, US) staining according to manufacture’s instructions. The percentage of apoptotic cells (Annexin V+/7-AAD- and Annexin V+/7-AAD+) was determined by using flow cytometry analysis (Beckman Coulter FC500).

### *In vitro* angiogenesis assay

The *in vitro* angiogenesis assay was performed using HREC at passages 2–4 as previously described^29^. Briefly, 2 × 10^4^ HRECs together with DMSO or tigecycline were plated onto the solidified complete Matrigel matrix (BD, Biosciences, US) in 96-well plate. The capillary network was formed after 6–8 h incubation. Photos were taken under light microscopy.

### Boyden chamber migration assay

HREC (4 × 10^4^/24-well plate) together with DMSO or tigecycline were seeded in the top of the insert in serum-free media and 10% FBS was placed in the lower chamber. After 4–6 h incubation, migratory cells moved through the pores toward the FBS below and were fixed, stained with 0.4% Giemsa (Sigma, US). The cells spreading on the upper surfaces of the filter (non-migrated cells) were wiped away with cotton swabs.

### Immunoblotting

Cells (2 × 10^6^/6-well plate) together with DMSO or tigecycline were incubated for 24 hours. Samples of cell lysate were prepared for NuPAGE electrophoresis according to the manufacturer’s instructions (Life Technologies, US), followed by electrophoresis and western blot analyses using antibodies against Cox-1, Cox-2, Cox-4 and β-actin (Cell Signalling, US). Immunoband intensities were analysed by Image 4.0 (BioRad).

### Real time-PCR

Cells (2 × 10^6^/6-well plate) together with DMSO or tigecycline were incubated for 24 hours. Total RNA were extracted from drug-treated cells using TRIzol-based reagent (Invitrogen, US) according to the manufacturer’s instructions. The first-strand cDNA was synthesized with the SuperScript First-strand Synthesis System (Invitrogen, US). Quantitative PCR was carried out using RT2 SYBR Green qPCR MasterMix on CFX96 RT PCR system (Bio-rad, CA). The sequence of primers for human Cox-1, Cox-2, Cox-4, MT-ND6, MT-CO2, and SDHA are the same as previously described^11,38^. The quantity of the transcript was normalized to the level of β-actin.

### Mito stress test assay

Cells (20,000/well in 24-well plate) were treated with drug for 24 hours. Oxygen Consumption Rate (OCR, indicative of mitochondrial respiration) was measured at 37^0^C in a CO_2_-free environment on a Seahorse XF24 extracellular flux analyser (Seahorse Bioscience, US) according to Seahorse Bioscience protocols. We measured the basal OCR, and then sequentially injected oligomycin, carbonyl cyanide-p-trifluoromethoxyphenylhydrazone and Antimycin A to assess the maximal OCR.

### Mitochondrial complex activities

Cells (100,000/well in 12-well plate) were treated with drug for 24 hours. Complex I, II and IV activities were assessed using total cell lysate and were determined using Mitochondrial Complex I, II and IV Activity Assay Kits (Novagen, US) according to manufacture’s instructions. The activity levels were indicated by the decrease in absorbance in mOD/min at 340 nm (I), 600 nm (II) and 550 nm (IV) in kinetic mode using Tecan Infinite200 Microplate Reader (Mannedorf, Switzerland).

### Cellular ATP and oxidative damage assays

Cells (100, 000/well in 24-well plate) were treated with drug for 24 hours. ATP levels were measured by ATPlite Luminiescent Assay kit (Perkin Elmer, US). DNA was extracted using the DNEasy Mini Kit (Qiagen, US). Oxidative DNA and protein damage was measured by quantifying 8-hydroxy-2′-deoxyguanosine (8-OHdG) levels using the OxiSelect Oxidative DNA Damage ELISA Kit (Cell Biolabs) and by quantifying protein carbonylation levels using the Protein Carbonyl ELISA Kit (Enzo LifeSciences), respectively. Lipid peroxidation was measured using the Lipid Peroxidation MDA Assay Kit (Abcam) following the manufacturer’s protocol. The absorbance was read on a Spectramax M5 Microplate reader.

### Retinoblastoma xenograft in SCID mouse

All animal experiment protocols were approved by the Institutional Animal Care and Use Committee of Wuhan University. Experiments were performed according to the guidelines provide by the Institutional Animal Care and Use Committee of Wuhan University. NOD/SCID mice were obtained from Shanghai Experimental Animal Centre, Chinese Academy of Sciences. 100 µl of Y79 cells suspended in 50%/50% PBS/Matrigel were subcutaneously injected into mice flank. 7 days after injection, once tumours were palpable, mice were treated with vehicle (20%/80% DMSO/saline) or tigecycline (60 mg/kg/d) by intraperitoneal injection (n = 10 per group). Tumour volume (tumour length × width^2^ × 0.5236) was measured two times a week using callipers. 30 days after injection of cells, mice were sacrificed, tumours were excised and frozen in −80 °C. Tumour frozen section slides were fixed with 4% paraformaldehyde (Sigma, US), stained with primary antibodies against CD31, HEL and 4-HNE (Cell Signalling, US), and then secondary antibody conjugated with horseradish peroxidase-DAB (3,3′-diaminobenzidine). The nuclei were stained with hematoxylin.

### Statistical analyses

The data are expressed as mean and standard deviation. Statistical analyses were performed by unpaired Student’s t test. In multiple comparisons, ANOVA was used for statistical analyses. Values were considered statistically significant at *P* < 0.05.

### Data availability

The datasets generated during and/or analysed during the current study are available from the corresponding author on reasonable request

## Electronic supplementary material

Supplementary File
