# Supplementary material for: Tigecycline as a dual inhibitor of retinoblastoma and angiogenesis via inducing mitochondrial dysfunctions and oxidative damage
Source: Sci Rep. 2018 Aug 6;8:11747. doi: 10.1038/s41598-018-29938-x (PMC6079108; doi:10.1038/s41598-018-29938-x)
Supplement: Supplementary file 1 — Supplementary File [file 41598_2018_29938_MOESM1_ESM.doc]

**Tigecycline as a dual inhibitor of retinoblastoma and angiogenesis via inducing mitochondrial dysfunctions and oxidative damage**

Ying Xiong, Wei Liu, Qian Huang, Jierong Wang, Yanjun Wang, Huijuan Li, Xuedong Fu

**Supplementary File**

**
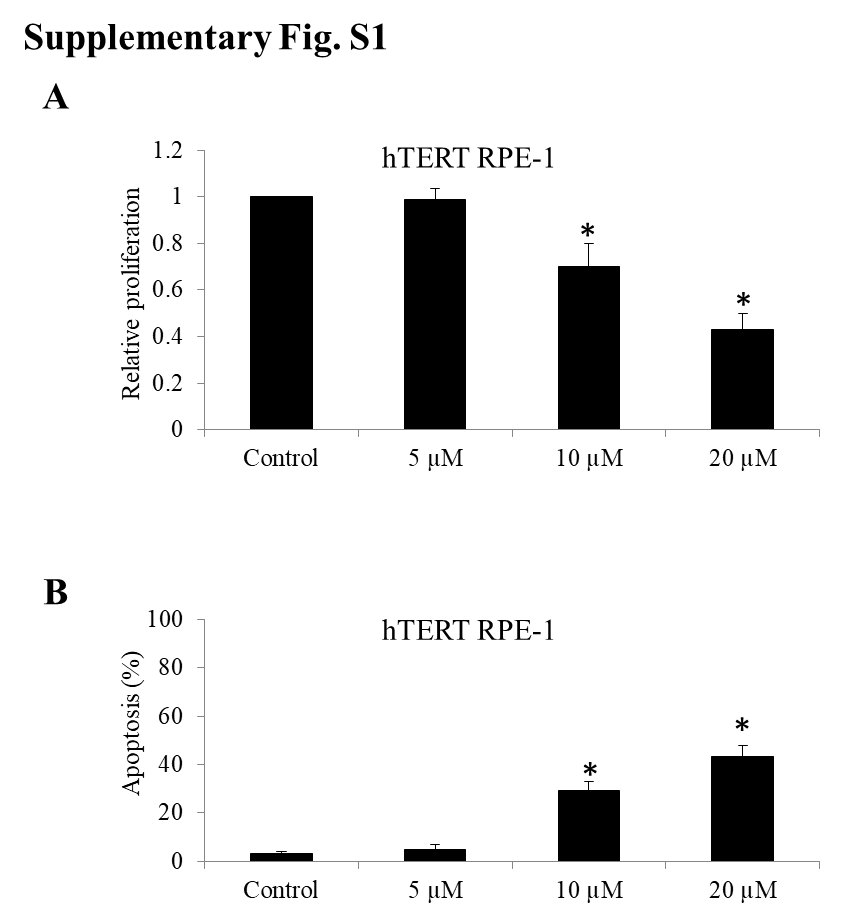
**

**Fig. S1. The inhibitory effects of tigecycline on human normal retinal pigment epithelial cells.** Tigecycline at 10 and 20 but not 5 µM significantly inhibits proliferation (A) and induces apoptosis (B) of hTERT RPE-1 cells. Cells were treated with tigecycline for 3 days prior to proliferation and apoptosis measurement. *, P<0.05, compared to control.

**
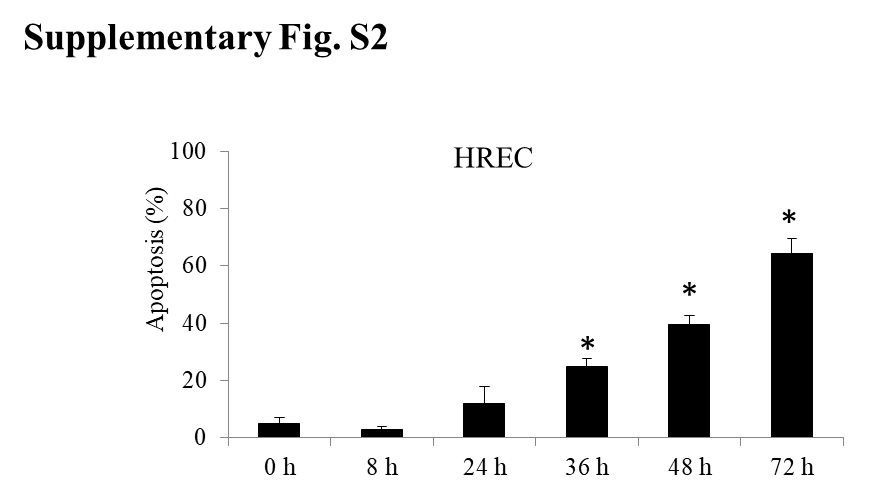
**

**Fig. S2. Tigecycline induces apoptosis of HREC in a time-dependent manner.** Tigecycline at 20 µM was used in the time course analysis. *, P<0.05, compared to 0 h.

**
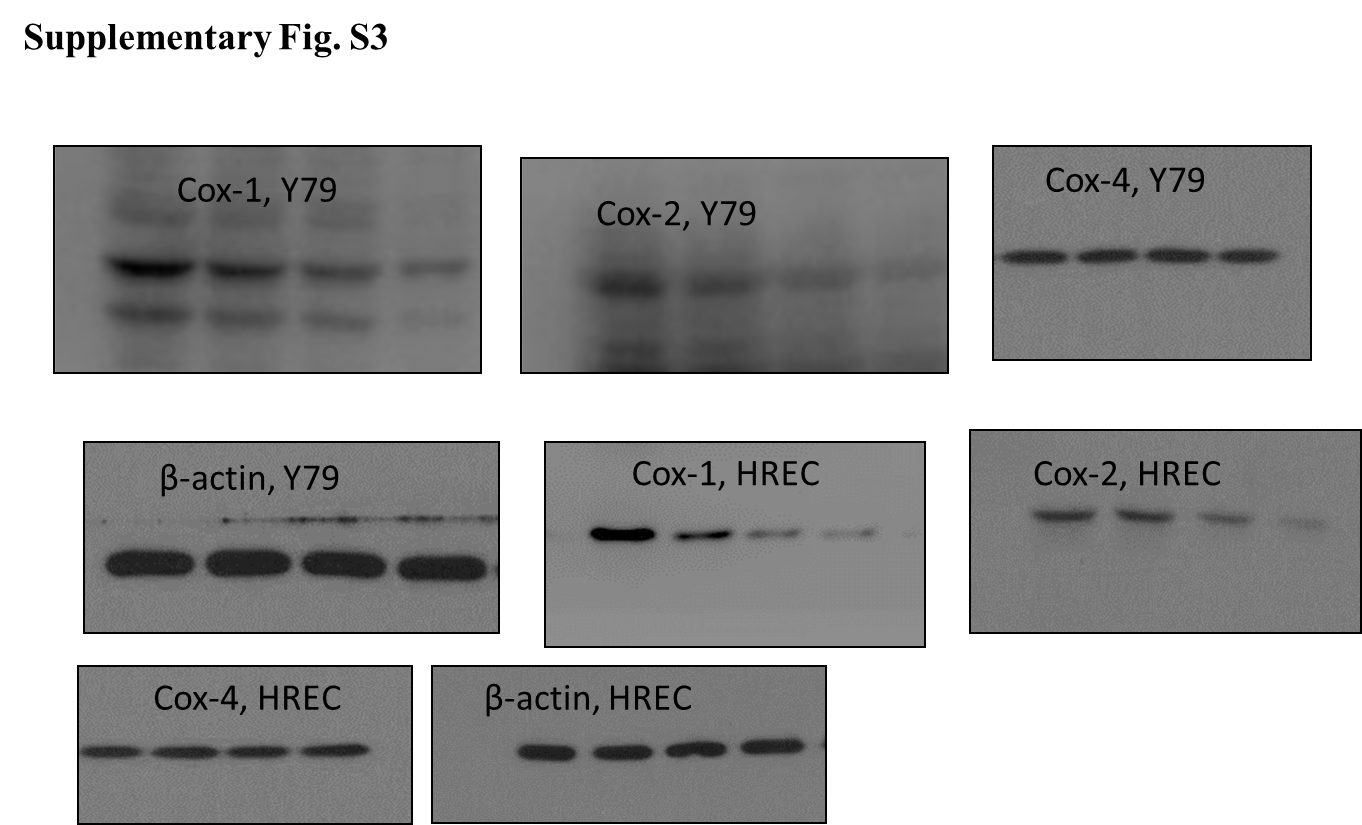
**

**Full unedited gels for Fig. 3A**

**
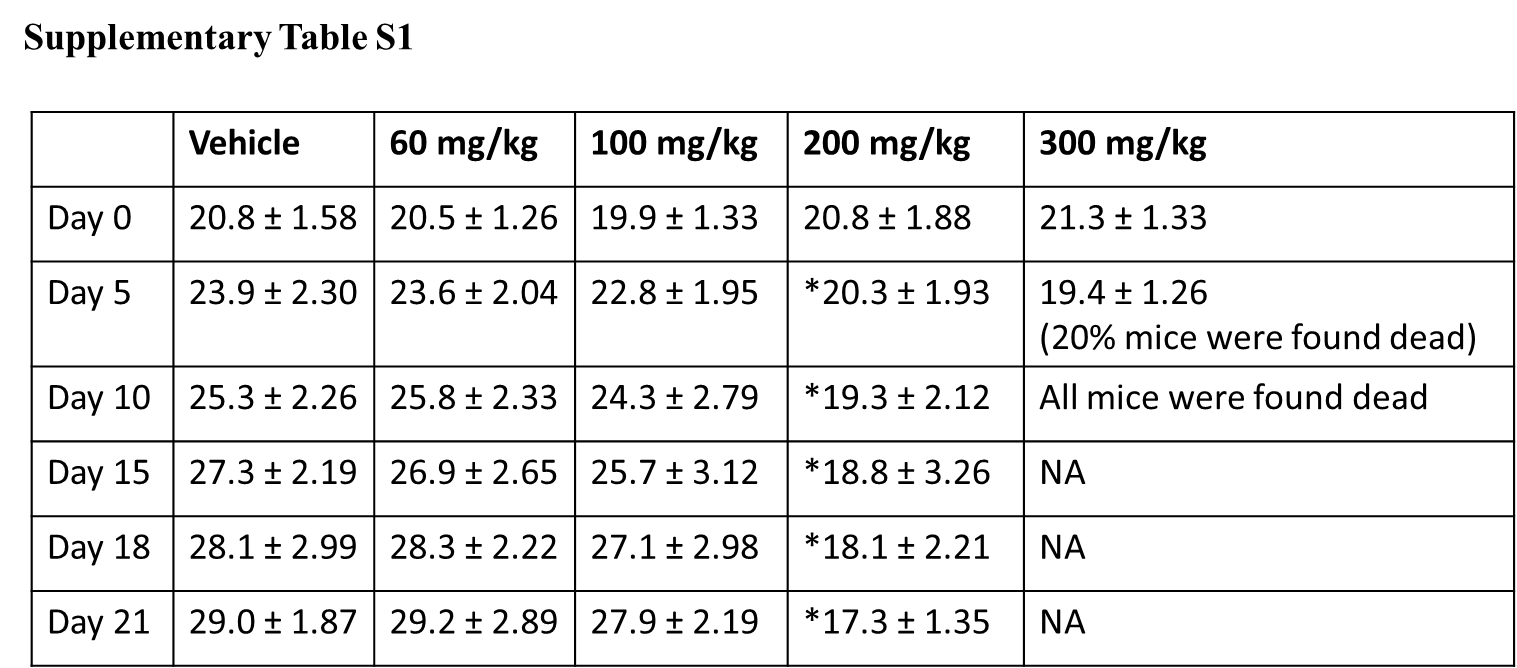
**

**Table S1. Mouse body weight in different groups treated with tigecycline.** Tigecycline at 60 and 100 mg/kg do not affect the mice body weight compared to vehicle control. Normal mice treated with tigecycline at 200 mg/kg has remarkable loss of body weight compared to control. All mice from 200 mg/kg group were weak with ruffled fur-coat, demonstrating the sign of toxicity. All mice from 300 mg/kg group were found dead within 10 days treatment. Each group has 10 mice. NA, not available. *, p < 0.05, compared to control.
